# Formal comment to Soler et al.: Great spotted cuckoo nestlings have no antipredatory effect on magpie or carrion crow host nests in southern Spain

**DOI:** 10.1371/journal.pone.0184446

**Published:** 2017-09-18

**Authors:** Daniela Canestrari, Diana Bolopo, Ted C. J. Turlings, Gregory Röder, José M. Marcos, Vittorio Baglione

**Affiliations:** 1 Department of Biology of Organisms and Systems (BOS), University of Oviedo, Oviedo, Spain; 2 Research Unit of Biodiversity (UMIB, CSIC, UO), Mieres, Spain; 3 FitzPatrick Institute of African Ornithology, University of Cape Town, Cape Town, South Africa; 4 Institute of Biology, University of Neuchâtel, Neuchâtel, Switzerland; 5 Dep. of Agro-forestry, University of Valladolid, Palencia, Spain; 6 Sustainable Forest Management Research Institute, Palencia, Spain; 7 Departamento de Biodiversidad y Gestión Ambiental, Universidad de León, León, Spain; Consiglio Nazionale delle Ricerche, ITALY

## Introduction

Replicating research is crucial to assess the generality of findings [1]. Yet, in ecology, the complexity of data collection and experimentation often precludes the possibility of going beyond single–population studies. The study by Soler et al. [2] is therefore most welcome, as it provides new insights on the possible role of great spotted cuckoo in protecting the nest of its corvid hosts. In a previous article [3], we suggested a mechanism based on the malodorous secretion of great spotted cuckoo chicks to explain why the presence of the parasite in the nests of carrion crows in northern Spain increased the probability of nest success (i.e. fledging at least one host chick) as compared to non-parasitized nests. Soler et al. found no evidence supporting an anti-depredatory function of cuckoo chicks in their studied populations and proposed an alternative mechanism that may explain our experimental results. Here we would like to address a) the differences between the results of the two studies and b) the proposed interpretation of our translocation experiment. We will also respond to the concerns raised by Soler et al. on some of the analyses presented in our paper.

## Antidepredatory effect of foul smelling cuckoo secretion

Soler et al. did not detect an anti-depredatory effect of cuckoo chicks for host nestlings in their study area. In the case of carrion crows, however, this may be explained by the very low predation rate of this species in the southern study site (7 nests predated during nestling phase in four years, out of 66 nests surveyed; 4 nests predated out of 59 during the translocation experiment) that would preclude detecting a possible protective effect of the parasite.

Soler et al. included in their study relevant data on another host species, the magpie *Pica pica*, which also seemed to gain no anti-depredatory benefits from being parasitized. However, previous experiments showed that repellence of great spotted cuckoo secretion varied markedly depending on the species of nest predator, with the carrion crow being the least sensitive [3]. This scavenging species also showed no response to the synthetic version of the great spotted cuckoo secretion [4] and to the natural secretion of the common cuckoo *Cuculus canorus* [5]. The carrion crow is the main predator of magpie nests in the population studied by Soler et al. [2,6,7], which might explain why no protective effect of cuckoo chicks was observed. Interestingly, a positive effect of the presence of cuckoo chicks on magpie nest success was reported in a different population in southern Spain (Sierra Morena) where carrion crows are rare and are not an important predator of magpie nests [8]. It can be argued that this might be a spurious effect caused by pooling data from incubation and nesting period, merely explained by cuckoo choice for safer nests at the egg stage [2]. However, in our own study area, magpie parasitized nests showed a higher success than non-parasitized nests when nestling period was considered alone (0.9 *vs* 0.64; Z = 2.12, n = 135, p = 0.034; result of a Generalized Linear Mixed Model with binomial distribution, where year was fitted as random factor). Unlike in the area studied by Soler et al., we found that magpies build most nests deep inside dense and thorny bushes that grant better protection [9] and are possibly inaccessible to crows.

Although replicated studies across species/populations cannot be regarded as validatory exercises because of the dynamic response of organisms to different environments [10], Soler et al. [2] expand our understanding of how the presence of a cuckoo chick can affect host survival. Indeed, the new data document a variation across host species and between populations that differ in ecological conditions. This exemplifies the importance of replicating studies for documenting the variability of ecological patterns, as we shall discuss at the end of this comment.

## Self or brood protection?

Soler et al. [2] raised further concerns on the suggested defensive effect of the malodourous secretions of great spotted cuckoo chicks on host chicks. They asserted that this secretion should be conservatively considered a self-protection mechanism, given that species like the common cuckoo, which is raised alone in the host nests, [5,11] as well as some other non parasitic cuckoo species [12] show similar chemical adaptations. We fully agree. Indeed, mutualism (relationship between individuals of two species where both benefit from the association) can arise as by-product of selfish traits [13], i.e. in absence of specific co-adaptations. Cuckoo selfish defence does not exclude the possibility of protection of the rest of the brood (or at least part of it) if, for example, a nest predator is deterred by the secretion and ends an attack before killing the entire brood. This may be particularly true for predators that raid a nest several times. They may be repelled and give up returning to the same nest to get more nestlings, therefore sparing some host chicks. It is also not necessary, as authors suggest, that the cuckoo chick is attacked first or that it sprays its secretion towards the aggressor to provide a benefit to host parents. As long as the cuckoo is not the last one to be attacked (i.e. after the rest of the brood has been already killed), the host will enjoy a benefit as compared to nests without a cuckoo. Finally, Soler et al. raise “*the question why cuckoo nestlings should protect the carrion crow nests mates*” when, in fact, they grow better alone [2]. We suggest that this side effect of the self-defensive mechanism may be a bearable cost if traded off with a reduction of the risk of being predated. Although Soler et al. raised important points, we think that an anti-depredatory function of great spotted cuckoo secretion remains, as we stated in our study [3], a *plausible* explanation for the detected causal positive effect of cuckoo chicks presence on nest success in our studied crow population. Further investigation, however, is certainly needed, particularly because Soler et al. proposed an alternative mechanism that deserves careful consideration, as we shall discuss below.

## Experimental design

Our long-term data on crow reproduction in northern Spain showed that parasitized nests had higher success (defined as at least one crow chick fledged) than non-parasitized nests. A translocation experiment was therefore designed to assess whether this effect was causally linked to the presence of the cuckoo chick (instead of, for example, being due to cuckoo female choice of the best host parents; please note that this experiment was not intended to test the putative protective function of cuckoo secretion). The experiment involved a positive control (adding/removing crow chicks) that addressed the effect of the manipulation. Soler et al. [2] suggest that the observed higher success of parasitized crow nests in our experiment could be due to a side effect of the protocol, where a) removal of the first and only chick in the nest (the cuckoo) might have been perceived by the crow host parents as partial nest predation, causing a reduction in their defence of the nest; b) addition of a cuckoo chick, which hatch precociously, grow feathers rapidly and beg vigorously, would have enhanced host nest defence by parents willing to protect nestling/s of high phenotypic quality. We believe that their explanation does not invalidate our conclusions, as we shall explain below, but represents an interesting and plausible non-exclusive alternative mechanism to the anti-depredatory effect of cuckoo chicks.

### Removal of cuckoo chick

Soler et al. assert that our experimental cuckoo chick removal must have invariably implied the loss of the first and *only chick in the nest for donor adult hosts*. This might have been perceived as partial nest predation, possibly driving a reduction in nest defence that would explain a higher failure rate. However, this is not what happened in our experiment. In our crow population, incubation begins with the second or third egg (maximum clutch size = 7), lasting 19.41 ± 0.03 days (N = 200), and cuckoo eggs are often laid after incubation has started [14]. This, combined with the fact that hatching failure of the first laid crow eggs is rare, causes an average (±SE) age advantage of cuckoo chicks over the *first* hatched crow chicks of 2.30 ± 0.31 days [14]. Indeed, in our experiment, nine out of sixteen donor nests had already at least one crow chick present at the moment of cuckoo removal. In contrast with the hypothesis of reduced nest defence by parents that lost their first chick, these donor nests did not fail less often than donor nests where the cuckoo was alone at the moment of translocation (failure rate = 0.67 and 0.71 respectively; n = 9 and 7; Fisher Exact Test, p = 1). The supposed “side effect” of the experimental protocol is therefore unlikely.

### Addition of cuckoo chick

Following the same rationale, Soler et al. [2] also propose that the presence of an early hatched, rapidly feathered and vigorously begging chick may increase adult nest defence, explaining why nests where cuckoos were added failed less often that nests without the parasite. First, it should be noted that cuckoo chicks were added to nests with at least one crow chick already present in 7 of 14 cases and that nest success was identical in nests with or without host nestlings at the moment of translocation (71.4%). Second, the potential mechanism suggested by Soler et al. does not dismiss the positive effect of the parasitic chick on survival on crow chicks. Indeed, under natural conditions only a cuckoo chick can hatch and grow feathers precociously. By synchronizing donor and receiver nests in relation to laying date, our experiment reproduced the natural event of parasitism and correctly tested the causal positive effect of the presence of a cuckoo chick on nest success, irrespectively of the mechanism behind it. Most importantly, given that raising a cuckoo does not decrease adult crow survival or future fecundity [3], the benefit of being parasitized remains, regardless of whether it is achieved through increased parental nest defence or cuckoo predator deterrence. It should also be noted that the two mechanisms are not mutually exclusive. Indeed, the parasite might boost the efficiency of its chemical weaponry by also manipulating parental behaviour. This hypothesis was not considered in our original paper and represents a valuable contribution by Soler et al. that deserves further investigation. Replicating the experimental protocol of Soler et al. in our study population might be a way to address the issue, as the authors suggest. Additional data on the occurrence of partial nest predation, which under the chemical defence hypothesis should be expected to be higher in parasitized nests as compared to non-parasitized nests, might also help to disentangle the two hypotheses. Unfortunately, this is not possible at present, because we lack direct information to differentiate partial predation from other causes of mortality.

## Data analysis

In their argumentation against a possible anti-depredatory function of cuckoo chicks, Soler et al. [2] cast doubts on the correlation between the difference in the mean number of crows fledged in parasitized and non-parasitized nests, and the annual failure rate of non-parasitized nests (i.e. the correlation shown in Fig 3 in [3]). However, this analysis is actually stronger if, as authors suggest, we restrict the sample to years of high parasitism (≥10%, N = 10; Spearman rank correlation = 0.87, t = 4.88, p = 0.001; range of parasitized nests per year = 6–24). The correlation is even stronger if we progressively remove years with fewer parasitized nests until the limit allowed by the test (N = 7; Spearman rank correlation = 0.96, t = 8.14, p < 0.001; range of parasitized nests per year = 8–24). An alternative analysis that tests annual crow productivity as a function of nest failure rate and parasitism status also confirmed that raising a cuckoo chick can be beneficial for crows. In a Generalized Linear Mixed Model with negative binomial distribution (to correct for data over-dispersion) run in R [3] with “glmmadmb” package [4], the number of crows fledged significantly depended on the interaction between annual nest failure rate and brood parasitism (Z = 2.020, p = 0.04), with parasitized nests becoming more productive than non-parasitized nests after a threshold of failure rate (0.65). The model included group size (Z = 3.55, p < 0.01), clutch size (Z = 1.7, p = 0.09), and Julian date (Z = -4.21, p < 0.01) as explanatory variables, and year as random term. The new analyses therefore confirmed the strength of the original results.

## Conclusions

Our paper [3] showed that the association between a brood parasite and one of its hosts can be beneficial for both under certain conditions. Regardless of the mechanism behind the positive effect of great spotted cuckoo chicks on crow nest survival, this unexpected form of by-product mutualism parallels findings in other systems, where the outcomes of the interaction between species are condition dependent [15,16]. Soler et al. expand our knowledge, showing how great spotted cuckoo/host interaction can vary also across populations and host species. This makes the co-evolutionary scenario even more complex than previously thought. We agree with Soler et al. that, to fully uncover this complexity, more data are needed across a wider range of ecological contexts (e.g. composition of predator guild) and host populations. At present, the two studies [2,3] represent a good example of how replicating research in ecology is fundamental to improve our understanding of the variation of natural processes [1,10].
